# The evolving use of measurable residual disease in chronic lymphocytic leukemia clinical trials

**DOI:** 10.3389/fonc.2023.1130617

**Published:** 2023-02-22

**Authors:** A. Fisher, H. Goradia, N. Martinez-Calle, PEM. Patten, T. Munir

**Affiliations:** ^1^ Division of Cancer Studies and Pathology, University of Leeds, Leeds, United Kingdom; ^2^ Department of Haematology, Leeds Teaching Hospitals National Health Service (NHS) Trust, Leeds, United Kingdom; ^3^ Department of Haematology, Nottingham University Hospitals National Health Service (NHS) Trust, Nottingham, United Kingdom; ^4^ Department of Haematology, Kings College Hospital National Health Service (NHS) Foundation Trust, London, United Kingdom; ^5^ Comprehensive Cancer Centre, Faculty of Life Sciences and Medicine, King’s College London, London, United Kingdom

**Keywords:** leukemia, lymphocytic, chronic, measurable, residual, disease, trials, B cell

## Abstract

Measurable residual disease (MRD) status in chronic lymphocytic leukemia (CLL), assessed on and after treatment, correlates with increased progression-free and overall survival benefit. More recently, MRD assessment has been included in large clinical trials as a primary outcome and is increasingly used in routine practice as a prognostic tool, a therapeutic goal, and potentially a trigger for early intervention. Modern therapy for CLL delivers prolonged remissions, causing readout of traditional trial outcomes such as progression-free and overall survival to be inherently delayed. This represents a barrier for the rapid incorporation of novel drugs to the overall therapeutic armamentarium. MRD offers a dynamic and robust platform for the assessment of treatment efficacy in CLL, complementing traditional outcome measures and accelerating access to novel drugs. Here, we provide a comprehensive review of recent major clinical trials of CLL therapy, focusing on small-molecule inhibitors and monoclonal antibody combinations that have recently emerged as the standard frontline and relapse treatment options. We explore the assessment and reporting of MRD (including novel techniques) and the challenges of standardization and provide a comprehensive review of the relevance and adequacy of MRD as a clinical trial endpoint. We further discuss the impact that MRD data have on clinical decision-making and how it can influence a patient’s experience. Finally, we evaluate how upcoming trial design and clinical practice are evolving in the face of MRD-driven outcomes.

## Introduction

The treatments used for chronic lymphocytic leukemia (CLL) have undergone significant changes in the last decade, from using chemoimmunotherapy (CIT), for example, fludarabine, cyclophosphamide, and rituximab (FCR), bendamustine and rituximab (BR), or chlorambucil (Clb)-based treatments such as Clb and obinutuzumab (CO), to fully embracing regimens inclusive of small-molecule-directed therapies (1–3).

This review focuses on the ability of CIT and targeted drugs alone or in combination to achieve deeper measurable residual disease (MRD) responses while acknowledging the strengths and weaknesses of the application of MRD utility in clinical and trial practice. The position of newer agents has been cemented in frontline therapy in the UK as reflected in the recent guidelines from the British Society of Haematology (BSH), European Society of Medical Oncology (ESMO), and National Comprehensive Cancer Network (NCCN) of the United States (4–6). Simultaneously, clinical trials have demonstrated how MRD correlates with the clinical endpoints of progression-free survival (PFS) and overall survival (OS) (7–14). MRD has been accepted as a surrogate endpoint that enables trials to report on outcomes sooner than traditional endpoints such as PFS and OS and has already been shown to correlate with superior PFS and OS in CIT regimens (10, 11, 15). We now need to evaluate whether MRD can also play this role in clinical trials involving small-molecule-directed therapies (8, 16). In this review, we consider the current evidence in MRD detection and trials involving small-molecule-directed therapies that report MRD outcomes and place the evidence in a clinical context to evaluate how new drugs and laboratory technology can be utilized for maximal patient benefit.

## Current guidelines

The recently updated BSH (2022), ESMO (2020), and NCCN (2022) clinical guidelines on disease assessment and treatment for CLL reflect the incorporation of small-molecule inhibitor-based treatments into clinical practice. The guidelines are very similar in their approach to disease assessment prior to treatment initiation, which follows the International Workshop on Chronic Lymphocytic Leukemia (iwCLL) guidelines (17). The initial screening for TP53 disruptions prior to each line of therapy and immunoglobulin heavy-chain variable region (IGHV) mutational status are recommended (4). Both TP53 disruptions (mutation and/or deletion) and unmutated IGHV (uIGHV) status, where ≤2% of the BCR is mutated, confer unfavorable prognosis disease and can direct therapeutic options (5, 6).

The BSH, ESMO, and NCCN guidelines outline therapeutic options. For patients with TP53 intact IGHV mutated disease, the treatment can be either CIT or a small-molecule inhibitor-containing regimen with the options of venetoclax (V), a BCL-2 inhibitor; Bruton’s tyrosine kinase inhibitors (BTKis) ibrutinib (I) and acalabrutinib (A); and the PI3k inhibitor idelalisib (Id). Zanubrutinib (Z) is now a licensed option in both the United States (6) and the European Union (18). BTKis and PI3kis can be referred to as “BCR-directed therapy” (19). Monoclonal antibodies can be used in combination with small-molecule inhibitors [the approved combinations vary depending on the global region (4–6)] and include rituximab (R) and obinutuzumab (O) (type I and II anti-CD20 monoclonal antibodies, respectively) (20). CIT regimens remain an option in specific settings including FCR and BR, but the use of CIT is very much reduced compared with previous guidelines (21) with the increased availability of effective non-CIT options frontline.

Comorbidities and the patient’s choice alongside TP53 and IGHV results form the backbone of therapy selection. TP53 must be intact for CIT to be an option (4–6). As frontline therapy, patients with intact TP53 and IGHV mutated disease (mIGHV) may benefit from FCR (12). TP53 intact fit patients can also be offered venetoclax–obinutuzumab (VO) or I if they have uIGHV disease. There is more flexibility of choice in the NCCN guidelines where Z and AO are preferred options in this fit group alongside VO (6). In less fit patients with intact TP53 and any IGHV status, VO, I, or A is recommended by the BSH and ESMO. AO is included in the recommended options by the BSH but is not currently reimbursed by the NHS in the UK. CO is an option in the ESMO guidelines if targeted therapies are contraindicated or not available. In addition, zanubrutinib is a preferred option for these patients in the NCCN guidelines. For patients with TP53 disruption, CIT is dropped from all guidelines. The treatment recommended for TP53-disrupted patients includes VO, I, or A in all aforementioned guidelines, with the additional options of AO or V by the BSH, IdR or V by the ESMO, and AO or Z by the NCCN. The potential benefit of IV combination therapy in uIGHV disease is mentioned in the BSH guidelines, and this combination can be used for uIGHV disease in the United States (4–6).

The choice of therapy for relapsed or refractory disease is more varied and depends on TP53 status, whether the patient is eligible for consideration of an allogeneic stem cell transplant, and which therapy the patient has received before. In the BSH guidelines, the agents including all those discussed for first-line treatment (except CIT), but without TP53 disruption, are mandated to be used, and venetoclax–rituximab (VR) and Id are options. The ESMO guidelines recommend considering the duration of remission, stating that for long remissions (>36 months), repetition of the frontline therapy used is an option. As with their frontline treatments, the NCCN also recommends Z in the relapsed and refractory setting. The sequencing suggestions for therapies consider previous treatment, with different agents preferred to those used as first-line. At present, neither the iwCLL guidelines nor any of the BSH, ESMO, or NCCN guidelines include the use of resistance mutation testing as part of treatment selection to determine the sequencing of treatment. Further discussion on the sequencing of therapy is beyond the scope of this review. Prior to the initiation of treatment, patients can be considered for a suitable clinical trial (4). At present, CAR-T cells and bispecific antibodies are only available as part of clinical trials in the UK.

Response assessment using MRD is discussed in the ESMO and NCCN guidelines, but not by the BSH. The iwCLL guidelines state that MRD can be assessed in patients in complete remission (CR) who have cleared peripheral blood for at least 2 months, thereby implying its use to be restricted to bone marrow assessment of patients in complete CR, but the wording is ambiguous, perhaps reflecting the relative infancy and unknowns in using MRD assessment as a response criterion compared with some of its other recommendations (17). The ESMO and NCCN guidelines reflect this position, stating the use of MRD in clinical trials (5) and that MRD may be a useful predictor of PFS (6), but that it cannot be used to inform treatment decisions at the present.

## Assessment of MRD

The technology to detect residual clonal malignant cells has evolved, alongside its nomenclature, from “residual disease” to “minimal residual disease” to our current term “measurable residual disease” (22) as set out by the International Steering Committee (ISC) on CLL MRD (22). The term “measurable” stresses the point that we need to look at the limit of detection (LoD) of the assay employed to interpret the result. In the quest for “how low can you go,” it is likely that even between accredited laboratories, some may be able to report this at 2 log_10_ (2 orders of magnitude) or lower than others. For undetectable MRD (U-MRD) to be compared across settings, this context becomes important (22).

A recent report by the ISC on CLL MRD (22) has helpfully defined MRD according to the proportion of residual CLL cells detectable: MRD4 is 1 in 10^−4^ (1 leukemic cell in 10,000 leukocytes or 0.01%), MRD5 is 1 in 10^−5^ (1 leukemic cell in 100,000 leukocytes or 0.001%), and so on (22). The current international consensus for “undetectable” MRD is U-MRD4, although some trials report data from MRD5 or lower (7). MRD4 has the potential to emerge as a reproducible prognostic stratification factor for PFS after treatment, particularly for the treatment with small-molecule inhibitors.

It is worth noting that LoD is different from the limit of quantitation (LoQ). For example, an assay may detect down to 10 cells, but it cannot be reliability quantified (for the purposes of reproducible and standardized reporting) until 50 cells of a phenotype are confirmed (23, 24). U-MRD4, “MRD4,” or 10^−4^ may also be reported for clinical purposes (for example, used in trial endpoints), when the assay can detect <10^−4^. The guidelines by Rawstron et al. (24) state that if the minimum reproducible population is 50, 500,000 events are required for an LoQ of 0.01%, but an LoD of 0.01% could be achieved on 200,000 cells (20 events). This becomes important when assessing the specificity of the flow cytometry marker combination to produce reproducible populations and considering the number of events (viable cells) required to evaluate post-treatment populations (which can make the timing of MRD assessment and the sample volume a practical consideration).

The current validated techniques for MRD assessment include multiparameter flow cytometry and real-time quantitative polymerase chain reaction (RQ-PCR) assays (9, 22, 24) (also known as reverse-transcriptase quantitative real-time PCR, RT-qPCR), with the addition of high-throughput sequencing (HTS) where available (25).

### Multiparameter flow cytometry

Flow cytometry has traditionally been used for MRD testing in CLL and requires a minimum of four markers to detect down to U-MRD4 for reporting the patient as “MRD negative” as per the iwCLL guidelines (17). The European Research Initiative in CLL (ERIC) (24–26) has published guidelines on obtaining this standard. There have been multiple studies using flow cytometry technology that aim to lower its limit of detection further. In practice, it is limited by the number of cells required for input, the number of colors that can be assessed by available laboratory flow cytometers, and the physics of fluorophore spectrum overlap. **Table 1** provides an overview of the LoD of flow cytometry marker combinations reported for CLL (24–29).

**Table 1:** Limit of detection reported for flow cytometry marker combinations.

Publication	Colors	Panel	Limit of detection	Events required
Rawstron et al., Leukemia, 2007 (26)	4	CD5/CD19 with one of CD20/CD38, CD81/CD22, or CD79b/CD43	≤1 × 10^−4^	1 × 10^6^
Rawstron et al., Leukemia, 2013 (24)	6	CD19/CD5/CD20 with either CD3/CD38/CD79b or CD81/CD22/CD43	6 × 10^−5^	0.5 × 10^6^
Rawstron et al., Leukemia, 2016 (25)	6	CD19/CD20/CD5/CD43/CD79b/CD81	1 × 10^−5^	2 × 10^6^
Rawstron et al., Leukemia, 2016 (25)	8	CD19/CD20/CD5/CD43/CD79b/CD81/CD22/CD3	1 × 10^−5^	2 × 10^6^
Dowling et al., Lab Medicine, 2016 (27)	8	CD19/CD20/CD5/CD43/CD79b/CD81/CD22/CD3	7 × 10^−5^	0.5 × 10^6^
Sartor et al., Clinical Cytometry, 2013 (28)	10	CD81/CD22/CD3/CD5/CD20/CD79/CD38/CD43/CD19/CD45	1 × 10^−5^	1.8 × 10^6^
Goshaw et al., Cytometry, 2021 (29)	14	CD79b, CD5, ROR1, CD23, CD19, CD81, CD20, CD200, CD40, CD43, CD38, CD3, CD10, CD45	1.1 × 10^−5^	1.5 × 10^6^

Rawstron et al., Leukemia, 2007 (26) was optimized to have an LoD of ≤1 × 10^4^, rather than defining the lowest LoD possible. The ERIC criteria state that the ideal white blood cell (WBC) for the test is >5 × 10^6^/ml (25).

The results outlined in **Table 1** demonstrate that obtaining an LoD of MRD5 (1 CLL cell detectable in ≤1 × 10^5^) is challenging, although many combinations have demonstrated an LoD between 10^4^ and 10^5^. A reliable detection of MRD at <1 × 10^−5^ is reported, but the number of events may have not been sufficiently reliable to report the median MRD at <10^−5^.

However, even in the absence of reliable median LoD <1 × 10^−5^, flow cytometry still provides the advantage that it can be combined with additional markers for diagnosis, to construct a single diagnostic/monitoring panel. Examples of these panels have included CLL-specific diagnostic markers such as CD200 or the MRD marker ROR1 (25, 30, 31) as part of extended panels. The 10-color flow cytometry used by Bazinet et al. (30) demonstrated this advantage, with >90% agreement with the ERIC panel results (24). Given that a limitation of flow cytometry is the assessment of sufficient viable cells, timelines of sample processing can make a significant difference. Therefore, the best flow results are obtained on samples <48 h old, and using the same assay for diagnosis and MRD assessment is likely to maintain sufficient throughput of samples to enable economical running of equipment within this timeframe. With these considerations in mind, flow cytometry even with inferior sensitivity (but wider availability) to newer methods could retain a place as a screening tool, enabling the selection of patients who appear MRD negative to have a more stringent assessment.

### DNA sequencing

RQ-PCR is recommended in the ERIC guidelines as an option for MRD assessment. The ERIC outputs have also reported on HTS using next-generation sequencing (NGS) technology with the clonoSEQ^®^ assay, which was assessed alongside multiparameter flow cytometry and demonstrated good concordance with flow cytometry results down to 10^−4^ (25). HTS is being performed in selected laboratories but requires quantitative validation (i.e., the number of sequencing reads to be correlated to the number of cells) (25). HTS does have the advantage of using DNA and, therefore, can be used on older samples or stored DNA samples, unlike flow cytometry, but this needs to be balanced with expense, increased operator and reporting time, and the data processing and storage facilities required (32). In **Table 2**, HTS has been considered here alongside the “current” techniques to demonstrate the comparison of sensitivity with RQ-PCR, and HTS is gradually being excepted as valid for MRD testing (9, 25, 26, 33–35).

**Table 2:** DNA sequencing techniques including RQ-PCR and NGS.

Publication	Technique	Primers	Sensitivity
Rawstron et al., Leukemia, 2007 (26)	RQ-PCR	BIOMED-2 and ASO	≤1 × 10^−5^
Böttcher et al., Leukemia, 2009 (9)	RQ-PCR	FR1/FR4 and ASO	2.2 × 10^−5^
Logan et al., PNAS, 2011 (33)	HTS	BIOMED-2	2 × 10^−5^
Raponi, BJHaem, 2014 (34)	RQ-PCR	FR1/FR4 and ASO	2.8 × 10^−5^
Rawstron et al., Leukemia, 2016 (25)	HTS	clonoSEQ^®^	1 × 10^−6^
Hengeveld et al., Blood, 2022 (35)	HTS	IGHV leader-based	≤1 × 10^−5^

Consensus primers such as BIOMED-2 or other FR1/FR4 primers can be used for the “preamplification” of the IgH VDJ region, prior to targeting with allele-specific oligonucleotide (ASO) primers in RQ-PCR.

### RQ-PCR

The RQ-PCR allele-specific oligonucleotide (ASO) assay uses primers designed for a germline framework region of IgH and an ASO patient-specific (junctional rearrangement), allowing malignant clone (patient)-specific targeted amplification. Some methods use ASO primers for both forward and reverse. One or more control genes are used to provide a measure for quantitation and quality (36). This requires a more specialized lab setup than flow cytometry, with the additional burden of a patient-specific primer design, but with a gain of sensitivity in some samples and good concordance with flow down to 10^−4^ but not reaching the 10^−5^ threshold (9, 34).

### HTS

Six-color flow cytometry was investigated alongside the commercially available clonoSEQ^®^ assay that sequences the BCR complementary determining region 3 (CDR3) using consensus primers to enable detection down to 10^−6^. Unlike flow cytometry, NGS is not a direct “number of events” and requires calibration and bioinformatics pipelines informing the quantification of results (how many sequences reflect the number of cells present), which makes interlaboratory standardization and validation challenging. NGS can also be influenced by PCR amplification bias (which can result in some sequences becoming preferentially amplified leading to their overrepresentation in results). Amplification bias can be identified as “expanded sequences” that are different from CLL-associated sequences and can be removed effectively by bioinformatics filtering, with Rawstron et al. (25) reporting a false-positive rate of 0.002% using clonoSEQ^®^. Logan et al. (33) used the BIOMED consensus primers and reported some bias in the PCR results. They also presented the design of a bioinformatics pipeline that could control for some of these errors, allowing the use of data for IGHV sequence reporting, which is used to define whether a case is mutated or unmutated, along with MRD reporting in the same assay (33).

Alongside increased sensitivity for HTS in comparison to flow cytometry techniques, HTS also allows the quantitation of >1 clonotype within the same assay, which is useful for multiple dominant clones or subclones in the context of clonal evolution.

HTS may deliver >10^−5^ to 10^−6^ sensitivity required for further MRD stratification when evaluating the response to CLL therapy, but it remains to be broadly standardized and accepted as a technique for MRD reporting (32).

## Clinical trials

MRD as a primary or secondary outcome of clinical trials has been at the forefront of recent trials involving small-molecule inhibitors. Here, we review trials where MRD outcomes have been included in the trial design, to evaluate the role that MRD plays and its relationship to traditional trial outcomes such as PFS and OS. The trials we have included are MURANO (37), CLARITY (38), ELEVATE-TN (39), CLL14 (40), CAPTIVATE (16), CAPTIVATE FD (41), GLOW (42), FLAIR (43), CLL13 (44), and GALACTIC (45).

**Table 3** provides a summary of the trials evaluated here including the drugs included in each arm and how MRD was included in the trial design.

**Table 3:** Summary of MRD inclusion in the trial design.

Trial	Arms	Phase	Treatment naive (TN) or relapsed/refractory (RR)	Fixed duration for non-CIT arms	Total participants	MRD as a primary outcome	MRD as a secondary outcome	MRD stopping rules
MURANO	BR *vs*. VR	3	RR	Yes	389	No	Yes	No
CLARITY	IV single arm	2	RR	No	54	Yes	No	Yes
ELEVATE-TN	A *vs*. AO *vs*. ClbO	3	TN	No	535	No	No^a^	No
CLL14	ClbO *vs*. VO	3	TN	Yes	432	No	Yes	No
CAPTIVATE MRD	IV then I or placebo if U-MRD4	2	TN	Yes	54	No	Yes, for the ibrutinib *vs*. placebo cohort	No^b^
CAPTIVATE FD	IV	2	TN	Yes	159	No	Yes	No
GLOW	IV *vs*. ClbO	3	TN	Yes	211	No	Yes, for both arms	No
FLAIR	FCR, IR, I, IV	3	TN	No	1,516	Yes, for IV *vs*. I or IR	Yes, for FCR *vs*. IR	Yes, for the ibrutinib-containing arms
CLL13	CIT *vs*. VR *vs*. VO *vs*. VIO	3	TN	Yes	926	Yes	No	No^c^
GALACTIC	O consolidation	2	TN or RR	Yes	48	Yes	No	No

A, acalabrutinib; B, bendamustine; CIT, chemoimmunotherapy; Clb, chlorambucil; FCR, fludarabine, cyclophosphamide, and rituximab; I, ibrutinib; O, obinutuzumab (also known as “G” in CLL13 and CLL14); R, rituximab; V, venetoclax.

^a^ELEVATE-TN and RR have MRD as an exploratory outcome.

^b^Randomized depending on U-MRD.

^c^Option to extend from 12 to 36 cycle duration if MRD positive.

### Relapsed and refractory disease

MURANO (37) and CLARITY (38) were the first trials of venetoclax combination treatments in CLL with MRD outcomes to report mature data in the relapsed and refractory (RR) setting. MURANO is a phase 3 trial of the combination of VR being compared with standard chemoimmunotherapy (bendamustine–rituximab, BR) (37). CLARITY is a phase 2 single-arm study of ibrutinib and venetoclax (IV) (38). **Table 4** provides a summary of these trials alongside the outcome data.

**Table 4:** Relapsed and refractory disease trial outcomes.

Trial	Median age/years	Regimen	Number recruited	ORR	CR/CRi rate	PFS	OS	U-MRD4 rate PB	U-MRD4 rate BM
MURANO	65	BR	195	67.7%	3.6%	36.3% at 24 m, median 17 m	86.6% at 24 m, 62.2% at 60 m	13.3% at 9 m	1.5% at 9 m
		VR	194	93.3%	8.2%	84.9% at 24 m, median 53.6 m	91.9% at 24 m, 82.1% at 60 m	62.4% at 9 m	27.3% 9 m
CLARITY	64	IV	54	89%	51%	Median PFS not reached	100% at 21.1 m	53% at 14 m	36% at 14 m

PFS and OS reporting statistics are reported after the duration stated on treatment.

BR, bendamustine–rituximab; VR, venetoclax–rituximab; IV, ibrutinib–venetoclax; m, months.

Three hundred eighty-nine patients with relapsed or refractory CLL who had previously received one to three previous lines of treatment (including at least one chemotherapy-containing regimen) were recruited. Previous bendamustine was allowed provided that patients had at least a 24-month duration of response. Patients were randomized in a 1:1 ratio, and this was stratified according to the 17p deletion status (TP53 deletion) and responsiveness to previous treatment. The primary endpoint was PFS, and the secondary endpoints included PFS among patients with 17p deletion, overall response rate (ORR), OS and MRD (to a sensitivity of 10^−4^), duration of response, and event-free survival (EFS). MRD status was assessed in peripheral blood with both ASO-PCR and flow cytometry and in bone marrow by flow cytometry.

#### PFS and OS

In MURANO, the 24-month PFS was significantly higher in the venetoclax–rituximab group *versus* the BR group (84.9% *vs*. 36.3%) (37), and PFS and OS remained significantly prolonged in the VR group at the 5-year follow-up, detailed in **Table 4** (46). This benefit was also reflected in the 17p deleted patients, with a 2-year PFS of 81.5% in VR compared with 27.8% in the BR arm (46). The success of VR is reflected as a choice of treatment in RR disease in many clinical guidelines (4–6).

In CLARITY, the primary endpoint was U-MRD4 after 12 months of treatment (17, 38), with the secondary endpoints of PFS, ORR (summarized in **Table 4**), and safety. Patients had been previously treated with at least one line of therapy that did not include I or V. Multiparameter flow cytometry was used for MRD assessment with a sensitivity of MRD5, and the MRD stopping criteria were included for those who also achieved CR/CRi as per the iwCLL criteria (17). The assessment of response used CT scans and peripheral blood (PB) and bone marrow (BM) MRD at 8, 14, and 26 months of treatment. Patients who were MRD negative in both PB and BM at 8 months were able to stop treatment at 14 months, and those negative at 14 months could stop at 26 months. Those negative at 26 months continued treatment with ibrutinib until progression but stopped venetoclax (38). The protocol was amended in 2019 for those not in U-MRD4 after 26 months to have venetoclax in addition to ibrutinib for a further 12 months (47).

The ORRs for both CLARITY and MURANO, alongside CR/CRi and MRD rates, are detailed in **Table 4**.

#### MRD outcomes

The primary MRD outcome analysis from CLARITY, detailed in **Table 4**, demonstrated that after 6 months, 14/50 evaluable patients were in U-MRD4 (47). This improved over time and 36% of patients achieved U-MRD4 in both BM and PB at the 14-month assessment (12 months of dual therapy, increasing to 44% U-MRD4 in BM at 26 months (38). This evidence informed the addition of trial arms I and IV in FLAIR, which uses a similar model of continuing treatment for twice as long as it took for the patient to become MRD negative (43).

Mature MRD data from MURANO and CLARITY have enabled the comparison of results with older trials including CIT. In MURANO, patients with a partial response on iwCLL criteria but who have U-MRD4 on peripheral blood had better survival outcomes than patients who have a complete response but are positive for MRD (10). Higher rates of clearance of MRD in the venetoclax arm were observed and were numerically higher than those reported for other agents in the relapsed/refractory setting [ibrutinib *vs*. bendamustine (48); FCR *vs*. FC (49)], with the caveat of intertrial comparison, and the MURANO results support the replacement of chemotherapy with venetoclax in the relapsed setting.

MURANO has also reported MRD kinetics, and longer-term survival outcomes were reported after 5 years of follow-up (46), outlined in **Table 4**. The 5-year OS was also significantly prolonged at VR than at BR. The analysis of outcomes by the MRD level demonstrated that those with U-MRD4 had superior OS in the VR arm, with survival at 5 years (EOT plus 3 years) of 95.3% compared with those with higher rates of MRD (72.9%). There was also a slower rate of MRD doubling time in the VR-treated patients compared with the BR-treated patients (46). The results demonstrate the durability of fixed-duration VR in the RR setting and raise the possibility of a treatment-free period for patients with relapsed/refractory CLL.

Longer-term MRD outcome data from CLARITY were presented at the American Society of Haematology Annual Meeting and Exposition (ASH) 2022. Of those in U-MRD4 at 6 months, this was sustained at either 26 or 38 months. Of note, 75% of this cohort had uIGHV disease, and the good rates of U-MRD4 and the durability of remission for U-MRD4 patients with IV have also been reflected in uIGHV patients in the later GLOW and FLAIR trials (47, 50, 51).

MURANO and CLARITY have provided safety and outcome reports prior to the data from several frontline therapy trials in CLL, including CAPTIVATE (16, 41), GLOW (42), FLAIR (43), and CLL13 (44). They also demonstrate a difference in regimen design between fixed-duration and continuous (BTKi) therapy, which is reflected in the latter trials and may direct how MRD assessment should be incorporated into response assessment.

### Treatment-naive fixed-duration trials

Most treatment-naive (frontline) trials fall into the category of fixed-duration treatments (for non-CIT arms). These include CLL14 (40), CAPTIVATE FD (16, 41), GLOW (52), and CLL13 (44), and all include venetoclax-containing arms (**Table 3**). Except for CLL13, all these trials have MRD as a secondary outcome (**Table 3**). The trial outcome data are summarized in **Table 5**.

**Table 5:** Treatment-naive fixed-duration traditional trial outcomes.

Trial	Median age/years	Regimen	Number recruited	ORR	CR/CRi rate	PFS	OS	U-MRD4 rate PB	U-MRD4 rate BM
CLL14	72	ClbO	216	NR	NR	Median PFS 35.6 m, 49.5% at 39.6 m	87% at 39.6 m	35% at 3 m post-treatment completion, 7% at 18 m post-treatment completion	56.9% at 3 m post-treatment completion
		VO	216	NR	NR	Median PFS not reached, 81% at 39.6 m	87% at 39.6 m	76% at 3 m post-treatment completion, 47% at 18 m post-treatment completion	17.1% at 3 m post-treatment completion
CAPTIVATE FD	60	IV	159	96%	56%	95% at 24 m	98% at 24 m	77% TP53-WT 81% TP53-D	62% TP53-WT 41% TP53-D
GLOW	71	IV	106	86.8%	38.7%	84.4% at 24 m, 80.5% at 30 m	90% at 27.7 m	54.7%	51.9%
		ClbO	105	84.8%	11.4%	44.1% at 24 m, 35.8% at 30 m	88.6% at 27.7 m	39.0%	17.1%
CLL13	61	CIT (FCR or BR)	229	NR	NR	NR	NR	52% at 15 m	37.1% at 15 m
		VR	237	NR	NR	NR	NR	57% at 15 m	43% at 15 m
		VO	229	NR	NR	NR	NR	86.5% at 15 m	72.5% at 15 m
		VIO	231	NR	NR	NR	NR	92.2% at 15 m	77.9% at 15 m

PFS and OS reporting statistics are reported after the duration stated on treatment.

ClbO, chlorambucil–obinutuzumab; VO, venetoclax–obinutuzumab; IV, ibrutinib–venetoclax; CIT, chemoimmunotherapy; FCR, fludarabine–cyclophosphamide–rituximab; BR, bendamustine–rituximab; VR, venetoclax–rituximab; VIO, venetoclax–ibrutinib–obinutuzumab; m, months.

CLL14 is a phase 3 trial comparing the long-term efficacy of fixed-duration regimes in previously untreated CLL (40). Four hundred thirty-two patients were randomized in a 1:1 ratio to receive 12 cycles of either venetoclax or chlorambucil both in combination with obinutuzumab for the first 6 cycles. The recruited participants required treatment by the iwCLL criteria (17) but were considered unfit due to coexisting conditions with a cumulative illness score of >6, a creatinine clearance between 30 and 69 ml/min, or both. Patients with a TP53 deletion or mutation could be included at the investigator’s discretion.

The primary endpoint of the study was investigator-assessed PFS. The secondary endpoints were independent reviewer committee-assessed PFS, ORR (assessed at 3 months after the end of treatment), CR rate, MRD response rate in PB and BM at the completion of treatment, duration of response, and best response achieved.

MRD monitoring was done for both PB and BM in CLL14, measured using ASO-PCR to a sensitivity of 10^−4^ with additional four-color flow cytometry and NGS in PB samples. BM samples were assessed in patients with treatment response at cycle 9 and 3 months after the end of treatment (EOT+3). PB MRD in the blood was assessed at baseline, cycles 7, 9, and 12, and subsequently every 3 months after EOT until 18 months and then every 6 months until 5 years post-randomization (40).

The CAPTIVATE FD cohort is a single-arm study of fixed-duration IV (**Table 3**). This cohort had a primary endpoint of CR rate, rather than disease-free survival (as in the CAPTIVATE MRD cohort), and U-MRD4 was a secondary endpoint (41).

Like CLL14, GLOW investigates a less fit population but explores IV as first-line treatment (42, 52). This is highly relevant to the practice of medicine in CLL, where the median age of diagnosis is 72 years (53). The participants were ≥65 years old or had comorbidities with a cumulative illness rating score of >6 or creatine clearance <70 ml/min. TP53 disruptions were excluded. The study was randomized 1:1 into two fixed-duration arms, IV (*n* = 105) or ClbO (*n* = 106) stratified by IGHV mutational status. Treatment was fixed duration with either 3 months of ibrutinib lead-in followed by 12 months of IV or 6 cycles of ClbO. PFS was the primary endpoint with U-MRD4 as the secondary endpoint. MRD was assessed by both 8-color flow cytometry and NGS (clonoSEQ^®^), with sensitivity down to MRD5, although endpoint reporting was set at U-MRD4.

The CLL13 trial (GAIA trial) evaluates the efficacy of venetoclax (and ibrutinib) in combination with an anti-CD20 antibody regimen (VO, VIO, VR) *versus* standard CIT (FCR or BR) (**Table 3**) in the first-line setting for fit patients requiring treatment for CLL. For patients in the VIO arm, 12 cycles were planned with the option to extend to cycle 36 in the presence of detectable MRD. Patients were randomized in a 1:1:1:1 ratio. Patients with TP53 disruptions were excluded given the known poor outcomes in this cohort with standard CIT (44).

The primary endpoints of CLL13 were the rate of U-MRD4 by flow cytometry in PB at month 15 (MO15) (VO arm *vs*. CIT) and PFS (VO *vs*. CIT). Evaluation of BM was also carried out at EOT+3 in patients achieving CR/CRi. The secondary endpoints included U-MRD4 rates, PFS for VIO and VR *versus* CIT, OS, and safety (44).

#### PFS and OS

The PFS and OS for CLL14, CAPTIVATE FD, and GLOW are given in **Table 5**, and CLL13 is yet to report these outcomes. In CLL14, there was a median follow-up of 39.6 months. The study found that patients in the VO arm had a significantly longer PFS than those in the ClbO arm with a superior 3-year PFS in the VO arm *versus* the ClbO arm (40). Interestingly, CAPTIVATE FD reported a CR/CRi rate of 56% which was the same for TP53 wild-type and disruption (*n* = 47) participants (41). GLOW demonstrated a superior PFS in IV *versus* ClbO after 24 months that was maintained at 30 months (**Table 5**) (52).

#### MRD outcomes

The end-of-treatment and interim MRD results for CLL14, CAPTIVATE FD, GLOW, and CLL13 are given in **Table 5**. CLL14 revealed the VO arm to have a higher rate of U-MRD4 in PB compared with the ClbO arm, with a higher rate of U-MRD4 in PB at 18 months after the end of treatment (**Table 5**). Patients with U-MRD4 after the end of either treatment had longer PFS compared with those with low detectable MRD or high MRD. OS was also shown to be longer in patients with U-MRD4. In the venetoclax–obinutuzumab arm, 8% of the patients showed an increase to low MRD and 20% to high MRD. The median time of conversion from undetectable to detectable MRD was not reached in the VO arm *versus* the 6-month conversion time in the CO arm. The rates of U-MRD4 were found to be higher in all risk groups in the VO arm. Deep remission was also found in patients with TP53 disruptions.

Residual disease kinetics in CLL14 revealed that it was rare for those who were found to have detectable MRD at the end of treatment to have had undetectable MRD at any timepoint, although the rates of U-MRD to detectable MRD conversion were not reported separately. This may prove to be useful in identifying patients who are at a higher risk of progression during/after treatment. The MRD monitoring of PB with NGS did show that some patients showed a deeper response of up to 10^−6^ in the VO arm. It was observed for the first time in a randomized study that the time to MRD conversion and eventual disease progression is longer with a deeper response. There was also reasonable concordance between the PB and BM MRD samples, suggesting that there may not be a need for regular bone marrow monitoring in the context of venetoclax and anti-CD20 fixed-duration therapy (40).

The U-MRD4 rates in CAPTIVATE FD showed higher rates of U-MRD4 in PB and BM after 12 months of IV, which was also reflected in the TP53-disrupted cohorts (**Table 5**). Interestingly, differences between TP53 wild-type and disrupted cohorts were not statistically significant (41), mirroring the CR/CRi results, and it is worth noting that the U-MRD4 rates were higher than the CR/CRi rates (**Table 5**). These MRD results are similar to CLL14 (40) after 12 cycles (12 months) of venetoclax (41).

In GLOW, the U-MRD4 rates were significantly higher for IV treatment in both PB and BM compared with ClbO (**Table 5**). There was also a higher concordance of MRD status in PB and BM in the IV group of 92.9%, compared with 43.6% for ClbO. In ClbO, patients who failed to achieve U-MRD4 were more likely to have disease progression, but this relationship was not seen in the IV arm (42). There was no significant difference in OS at the time of primary analysis (42).

MRD5 was reported using NGS in GLOW, at 40.6% in BM after IV and 7.6% after ClbO, with 90.9% concordance between PB and BM in IV participants. This represents the achievement of MRD5 by 79.3% in PB and 78.2% in BM of patients who had U-MRD4 in IV. U-MRD5 in PB was sustained in 80.4% of IV patients, compared with 26.3% of ClbO patients (52). The degree of concordance between PB and BM is encouraging for using PB to monitor MRD, and the relationship of U-MRD4 and U-MRD5 to PFS will be interesting data when it becomes available. There is evidence from the French Innovate Leukaemia Organisation (FILO) group trials that low-level MRD positivity at <U-MRD4 may be associated with inferior PFS compared with U-MRD5 (assay sensitivity 0.7 × 10^5^) (7).

Furthermore, GLOW has reported on residual disease kinetics after the EOT stratified by IGHV mutational status (50). In theory, uIGHV disease should respond well to ibrutinib-based therapies due to the dependence of this disease on BCR signaling (51), and this has been demonstrated in single-agent ibrutinib trials (54, 55). In the IV arm, the U-MRD4 rates at cycle 6 (6 months of treatment) in uIGHV disease were 52.2%, with EOT+3 U-MRD4 59.7%, and 77.6% of these patients retaining U-MRD4 at EOT plus 18 months (EOT+18). By comparison, mIGHV U-MRD4 rates at cycle 6 and EOT+3 U-MRD4 rates were 31.3% and 40.6%, respectively. However, if U-MRD4 was achieved at EOT+3, U-MRD4 was sustained in 80% of uIGHV patients and 76.9% of mIGHV patients (50). In the ClbO arm, just 12.2% of patients sustained U-MRD4 at EOT+18 (51). These findings reflect the durable responses seen in CLARITY, where 75% of the cohort had uIGHV (47), and demonstrate that while the addition of venetoclax increases U-MRD4 responses, the BTKi base still holds the preference for response in uIGHV disease.

In CLL13, the rate of U-MRD4 at 15 months was significantly higher in VO and VIO compared with CIT; however, the VR had comparable rates to CIT (**Table 5**) (44). The VO results are similar to those achieved in CLL14 at EOT (12 months), at 86.5% and 76%, respectively. The combination of VIO achieved higher U-MRD4 rates than dual IV or VO combinations and provided an exciting avenue for combinations that may help achieve deeper and more durable responses. Follow-up is still ongoing for this study and it will be interesting to compare the PFS with the high rates of U-MRD4 achieved (44).

### Treatment-naive continuous therapy trials

ELEVATE-TN is a phase 3 trial comparing the efficacy of A *versus* AO *versus* ClbO in patients with previously untreated CLL (**Table 3**) (39). Patients between the ages of 18 and 65 with comorbidities (creatinine clearance 30-69 ml min or cumulative illness score of >6) or those aged >65 were eligible for recruitment and were randomized in a 1:1:1 ratio. Crossover was allowed to the A monotherapy arm for patients who progressed in the ClbO arm. MRD was assessed by multiparameter flow cytometry in patients with CR/CRi, with a cutoff of U-MRD4.

The primary endpoint of ELEVATE-TN was PFS comparing AO *versus* ClbO. The secondary endpoints included PFS comparison between A and ClbO, ORR, time to next treatment (TTNT), and OS. The exploratory outcomes included the proportion of patients with U-MRD4.

CAPTIVATE MRD that focuses on untreated patients less than 70 years old is different from CAPTIVATE FD because it randomizes those in U-MRD4 to MRD-guided treatment discontinuation of IV. After 3 cycles of ibrutinib followed by 12 cycles of IV, participants who had U-MRD4 were randomized to either continue ibrutinib or receive placebo (double-blinded). Those who did not reach U-MRD4 were randomized to either ibrutinib monotherapy (I) or IV. In total, 164 participants were randomized with a median age of 58 years. The primary endpoint was disease-free survival at 12 months post-randomization for the I *versus* placebo cohort. The secondary endpoints were U-MRD4 rates, ORR, and safety (16).

FLAIR is the largest trial included in this review, with 1,516 patients randomized (**Table 3**). The primary endpoint is PFS as per the iwCLL criteria (56) to assess whether ibrutinib and rituximab (IR) are superior to FCR (57). In 2017, two additional arms were successfully added, comprising I and IV (43), supported by evidence from CLARITY (38). With the addition of the I and IV arms, the further primary endpoints are IV *versus* FCR PFS and IV *versus* I *versus* IR MRD negativity rate at 24 months post-randomization (43). The secondary endpoints for the trial include OS, rates of U-MRD4 (56), treatment response, quality of life (QoL), and cost-effectiveness (43, 57).

The patients recruited are between 18 and 75 years old, requiring therapy as per the iwCLL criteria, and fit for treatment using FCR. The high-risk (TP53 disruption) arm of I *versus* IV remains open. The IR arm was closed in 2018 in favor of I as the ongoing control, due to evidence that IR was not superior to I (58).

FCR was given for 6 cycles and then stopped. IR was given as 6 cycles of rituximab as per the FCR schedule, plus daily ibrutinib for 6 years or until the MRD stopping rules were achieved. I and IV were given as dual therapy for 6 years or until the MRD stopping rules were achieved. For all arms, therapy was ceased on progression or if the patient withdrew. Unlike other trials containing venetoclax, if MRD stopping rules are not achieved, venetoclax is given continuously for the trial duration alongside ibrutinib.

MRD is assessed throughout the FLAIR trial, in the PB from 3 months after rituximab for the FCR and IR arms, a BM and PB sample at 9 months post-randomization, and then in PB 6-monthly from 12 months for all arms, which allows dynamic evaluation of whether patients meet the MRD stopping rules. If a participant’s PB becomes MRD negative, they have a repeat MRD sample 3 months later. If this is negative, they have a PB sample and a BM sample 3 months later. If both are negative, they receive treatment for the same length of time as it took for them to become MRD negative, and then they stop (assuming they have remained MRD negative). Given the 6-year duration of the trial, participants becoming MRD negative at >3 years post-randomization would not be eligible to stop and would receive the full 6 years of treatment. The earliest treatment could stop at 2 years post-randomization. MRD testing continues 6-monthly for participants who have stopped treatment, and if they become MRD positive, they restart their treatment (43, 57).

The trial outcomes of ELEVATE-TN, CAPTIVATE MRD, and FLAIR are given in **Table 6**.

**Table 6:** Treatment-naive continuous therapy trial outcomes.

Trial	Median age/years	Regimen/arm	Number recruited	ORR	CR/CRi rate	PFS	OS	U-MRD4 rate PB	U-MRD4 rate BM
ELEVATE-TN	70	A	179	86%	1% at 24 m, 11.2% at 48 m	87% at 24 m	95% at 24 m, 87.6% at 48 m	7%^a^ at CR/CRi, 10% at 48 m	0%^a^
		AO	179	94%	13% at 24 m, 30.7% at 48 m	93% at 24 m	95% at 24 m, 92.9% at 48 m	49%^a^ at CR/CRi, 38% at 48 m	61%^a^
		ClbO	177	79%	5%	47% at 24 m	92% at 24 m	61%^a^ at CR/CRi, 9% at 48 m	10.9%^a^
CAPTIVATE MRD	58	IV	164	97%	46%	≥95% at 30 m	NR	75% at 15 m	68% at 15 m
FLAIR	70	FCR	385	NR	NR	67 m	92% at 52.7 m	NR	NR
	NR	IR	386	NR	NR	Not reached	92% at 52.7 m	NR	NR
		I	138	NR	8% at 9 m	NR	NR	0%	0%
		IV	136	NR	59.6% at 9 m, 93.4% at 24 m	NR	NR	71.3% at 24 m	65.4% at 24 m

PFS and OS reporting statistics are reported after the duration stated on treatment.

A, acalabrutinib; AO, acalabrutinib–obinutuzumab; ClbO, chlorambucil-obinutuzumab; IV, ibrutinib–venetoclax; FCR, fludarabine–cyclophosphamide–rituximab; IR, ibrutinib–rituximab; I, ibrutinib; m, months.

^a^In ELEVATE-TN, MRD was measured in participants with CR/CRi, and the percentage reflects the CR/CRi cohort only.

#### PFS and OS

The median PFS in ELEVATE-TN at a median follow-up of 28.3 months was significantly longer with AO *versus* ClbO (not reached *vs*. 22.6 months) and not reached in the A monotherapy arm, with the 24-month interim analysis data shown in **Table 6**. The acalabrutinib groups showed favorable PFS in the subgroup analyses over ClbO: AO *versus* ClbO in uIGHV patients (91% *vs*. 31%), del 17p (88% *vs*. 22%), del 11(q22.3) (87% *vs*. 24%), bulky disease (90% *vs*. 28%), and mutated *TP53* (95% *vs*. 19%). In TP53-disrupted patients, the 48-month PFS was 74.8% in the AO arm *versus* 76.2% in the A arm and 85.7% and 77.1%, respectively, for uIGHV patients. OS rates at 48 months were not significantly different between the arms (**Table 6**), and the CR/CRi rate increased up to 48 months with continuous acalabrutinib therapy in the AO and A arms (**Table 6**) (59). These results demonstrate favorable PFS in the acalabrutinib-containing arms compared with ClbO, which was increased further on high-risk subgroup analysis.

CAPTIVATE MRD recruited participants with a median age of 58 years and the ORR was 97% (17). The U-MRD4 cohort who received placebo and the CAPTIVATE FD cohort achieved rates of ≥95% PFS at 24–30 months (41) (**Table 6**). However, these are young and relatively fit patients, so additional U-MRD data (at MRD5 or MRD6) and longer-term follow-up for PFS and OS rates will be eagerly awaited.

FLAIR reported the analysis of the IR *versus* FCR arms at ASH in 2021 and demonstrated a significant difference in progression-free survival in favor of IR. Median follow-up was 52.7 months, with the median not reached for IR (**Table 6**). There was no difference in OS between the arms, which may in part be due to the FCR cohort progressing onto second-line treatment (60). Interim of the first 274 patients on I or IV reported a similar ORR at 9 months, but with a higher CR of 59.6% *versus* 8% favoring IV. Extending this to 24 months, 93.4% on IV were in CR (**Table 6**) and 39.7% had stopped treatment as per the MRD stopping rules (61).

#### MRD outcomes

The rates of U-MRD4 in ELEVATE-TN were similar in PB and BM in the patients with CR/CRi in the AO and ClbO arms (**Table 6**). Only one patient in the acalabrutinib monotherapy group had U-MRD4 in peripheral blood by flow cytometry, which is consistent with what has been described for other BTKis (61). Interestingly, there are similar rates of U-MRD4 in the two primary groups in patients achieving CR within each group, though a higher number of patients achieved CR in the AO arm. At the last two time points for MRD measurement in the 48-month follow-up, the U-MRD4 rate trends suggest U-MRD4 rates being sustained in AO and A and but dropping (61% to 9%) in ClbO (**Table 6**), although the sample size for these data is smaller than the overall study because MRD was only assessed in patients with CR/CRi (59). It is difficult to compare the U-MRD4 rates of this trial to others trials because MRD in CR/CRi only was assessed, whereas other trials such as CAPTIVATE (FD and MRD cohorts) have shown that U-MRD4 may occur in PR (16, 41).

After 12 months of IV in CAPTIVATE MRD, 75% of the patients achieved PB U-MRD4 and 68% BM U-MRD4 (**Table 6**). There was some correlation between CR and U-MRD4 achievement, with CR participants achieving 83% PB and 80% BM U-MRD4 and PR participants achieving 72% PB and 61% BM U-MRD4 (16). As seen in other trials of continuous BTKi therapy, such as ELEVATE-TN (59), U-MRD rates improved with the duration of therapy, with some participants entering the “not confirmed U-MRD” cohort as U-MRD4 in PB but not in BM then achieving BM U-MRD4 post-randomization. PB U-MRD4 in the MRD not confirmed arm remained unchanged at 45% but BM increased from 32% to 42% on ibrutinib and 31% to 66% on IV. The compartmental concordance of U-MRD4 in PB and BM was 94% (16). In the placebo arm, 84% of the patients remained in U-MRD4 after 12 months.

Interim analysis of MRD for the first 274 patients reaching 2 years post-randomization in the I *versus* IV arms of FLAIR was presented at the European Society of Haematology (EHA) congress and ASH in 2022. High rates of U-MRD4 were achieved in the IV arm by 24 months (**Table 6**), with no patients achieving MRD negativity with I (61), reflecting other trial arms using IV and BTKi monotherapies (**Tables 4**–**6**).

Further interesting results from FLAIR 2 years post-randomization were presented at ASH 2022 (51) for the IV arm, demonstrating a higher rate of U-MRD4 in uIGHV than mIGHV disease. The arms were well balanced for other disease variables when stratified by IGHV mutational status. In bone marrow at 2 years, 79.7% of uIGHV patients were MRD negative, with 53.1% of the patients becoming MRD negative by 9 months post-randomization. In patients with mIGHV disease, 56.4% were MRD negative at 2 years, with 34.5% being MRD negative at 9 months. The odds ratio was significant (95% CI: 1.59, 8.15) in favor of uIGHV. Similar trends were reflected in the peripheral blood. Of note, there was a lower rate of MRD negativity in subset 2, with 37.5% being MRD negative in bone marrow at 2 years post-randomization (51). These results reflect IGHV-stratified response analyses from GLOW (50).

These results are encouraging for IV providing excellent U-MRD4 response rates in the first-line treatment setting, a result that affirms the data from CAPTIVATE (MRD and FD) (16, 41) and the RR cohorts in CLARITY (47) and GLO W (52). IV has the potential to join VO as an approved first-line treatment for CLL in the UK (currently under NICE appraisal) and Europe and to move to a “preferred” option for certain patient cohorts in the United States (4–6).

### Treatment consolidation

The GALACTIC trial (45) investigated the effect of consolidation with obinutuzumab on MRD in patients who have received chemotherapy for CLL. Patients were eligible for this trial if they had received CIT in the last 3-24 months, had a WHO performance status of 0 or 1, and had no lymph nodes >1.5cm, and 48 patients in total were recruited.

MRD negativity in GALACTIC was set at U-MRD4, with MRD-negative patients (*n* = 19) monitored as a control group. The MRD-positive patients were randomized to receive obinutuzumab consolidation (*n* = 14) or not (*n* = 15). The primary outcome was the achievement of MRD negativity in patients who received consolidation. The secondary objectives were PFS, OS, and treatment-free survival (TFS). MRD was assessed at 6 months in BM and at 6, 12, and 24 months in PB. The results are summarised in **Table 7**.

**Table 7:** Treatment consolidation trial outcomes.

Trial	Median age/years	Regimen	Number recruited	ORR	CR/CRi rate	PFS	OS	U-MRD4 rate PB	U-MRD4 rate BM
GALACTIC	69	MRD-negative monitoring (no treatment)	19	NA	NA	NA	–	NA	NA
		MRD-positive monitoring (no treatment)	15	NA	NA	26.7% at 24 m	80% at 27.3 m	NA	NA
		MRD-positive O consolidation	14	NA	92.9%	92.9% at 24 m	71.4% at 24.9 m	92.8% at 6 m	71.4% at 6 m

PFS and OS reporting statistics are reported after the duration stated on treatment.

O, obinutuzumab; m, months; NA, not applicable.

Out of 14 MRD-positive patients randomized to the consolidation arm, 10 achieved U-MRD4 by flow cytometry in BM and 13 in PB. Though only a small cohort of patients was recruited, a significantly better PFS in patients who received obinutuzumab consolidation [*p* = 0.001; hazard ratio (HR) 0.21, 95% CI 0.07–0.67] was demonstrated, which matched the MRD-negative monitoring (control) group. There was a median follow-up of 24.9 months for consolidated patients and 27.3 months for controls, and over this period, there was no statistically significant difference between OS and TFS, although trends in data suggested improvement in both OS and TFS, but this evaluation was of limited sample size and follow-up duration (45). The cohort studied was heterogeneous in terms of prior therapy received and MRD level in the MRD-positive arm, but the arms were well balanced for key characteristics, for example, prior use of rituximab, considering the small cohort size. GALACTIC demonstrates that there is potential for MRD-driven consolidation treatment in CLL and that obinutuzumab could improved or be non-inferior (and safer) when compared with MRD rates reported after alemtuzumab consolidation (62–65).

## Considerations and controversies

### Emerging techniques for MRD evaluation

#### Digital droplet PCR

In digital droplet PCR (ddPCR), one DNA or cDNA copy is partitioned into a single droplet. After the amplification of thousands of droplets, the number of droplets containing amplified products is counted by the machine, to produce a quantification, e.g., 1 per 10,000. Unlike RQ-PCR, no calibration curves are required. Therefore, for small amplicons <150 bp, this provides efficient quantification. For variant allele or MRD detection, ddPCR has reached sensitivities of 1 × 10^−5^ for mantle cell lymphoma, hairy cell leukemia, and multiple myeloma, with improved sensitivity compared with RQ-PCR in these diseases (66). In CLL, ddPCR can detect *TP53* mutations down to 5 × 10^−5^ (67) and NOTCH1 down to <1 × 10^−6^ (68). At this level of sensitivity, ddPCR becomes a realistic option to detect MRD between 10^−4^ and 10^−6^, like NGS. The advantage of ddPCR could be time and computational efficiency, if a reliable and sensitive target can be determined. This is easier for standard mutations, for example, single-base changes that are consistent between patients, than it is for IgH variable mutations that are unique to the patient and currently require personalized primers. Multiplexing is also increasingly possible for ddPCR, increasing its application potential (69). Efficient workflow and the limits of sensitivity possibly make ddPCR a realistic option for standard MRD measurement in the future.

#### Cell-free DNA

Cell-free DNA (cfDNA) is a potentially useful CLL because the amount detectable has been shown to correlate with disease burden in all compartments, i.e., blood and lymph node, and will reflect nodal disease burden in small lymphocytic lymphoma, which may be underrepresented on conventional blood analysis, and overall disease burden as represented by a shift-in-compartment model of ibrutinib treatment initiation. However, it has not been compared with more sensitive MRD techniques such as NGS and ddPCR, although the results do correlate with standard flow cytometry MRD analysis (70). Mutational analysis of IgH, known genes, and clonal evolution can also be tracked by cfDNA. Whether the evaluation of the burden of disease in non-PB or BM compartments will enhance the stratification of disease prognosis in the era of small-molecule inhibitor therapy remains to be fully evaluated. This question, alongside the LoD of cfDNA, will determine whether cfDNA will become a useful MRD tool for CLL.

#### Mass spectrometry

Mass spectrometry is mostly used in the detection of single nucleotide variants, and its use is reported mainly in the context of myeloid malignancy (71). In the absence of characteristic point mutations in CLL cases, mass spectrometry is unlikely to be useful in the context of MRD. Detection of clonal point mutations including the ones correlating to drug resistance is possible with mass spectrometry, but it does not hold much advantage over NGS-based approaches, which require similar sample preparation, expertise, and equipment availability to achieve the same goal, with a wider application to *de-novo* variant, and complex (IGHV) mutational information. Therefore, the scope of this technology within the context of CLL is limited compared with the other options.

#### Cytometry time-of-flight

Cytometry time-of-flight (CyTOF) uses antibodies conjugated to metal isotopes and mass spectrometry techniques to determine the time of flight, which is related to the mass of the markers present in a cell. Like flow cytometry, it is a form of single-cell analysis, but can utilize more markers per cell given that it is not limited by fluorophore spectrum overlap (72). It can use more colors and fewer cells to reach a conclusion, with automated bioinformatics pipelines to alleviate the downstream data processing burden (73). However, it has not been trialed in CLL MRD at the time of writing and has been discussed more in the context of myeloid malignancy diagnosis and MRD monitoring. This technology is slow compared with flow cytometry, and with the advent of ddPCR and NGS-based methods that are already becoming integrated with current laboratory technology, it would be unsurprising for CyTOF to remain solely a research tool in the context of CLL.

#### Computational techniques

With the increased emphasis on MRD in clinical trials being likely to translate to increased demand for CLL MRD reporting in the future, the laboratory capacity to evaluate this workload efficiency becomes increasingly important, especially in healthcare systems with limited economic resources. Computational techniques to automate some processes could provide an answer, at least enabling efficient screening and reporting of simple cases, so clinical scientists can focus on more complex cases.

Goshaw et al. (29) developed a 14-color panel with the potential for automation separation using the Barnes–Nutt stochastic neighbor embedding (bh-SNE) analysis. There was an excellent agreement with traditional manual gating reporting for MRD. This approach has the potential to speed up the workflow and increase the interlaboratory consistency of reporting. The current pitfall is that few labs have a flow cytometer capable of assessing 14 colors, although the authors do comment that there was some possible redundancy of markers, and it is likely that a panel for automated reporting could be validated for 8-12 color cytometers (29). Artificial intelligence (AI) algorithms have been involved in gating for flow cytometry, which could reduce operator time (74).

Fully computational MRD assessment could become possible for some cases, which will save reporting burden if all CLL cases are screened regularly (75). Techniques such as NGS are already subject to established rigorous bioinformatics methodology. Whether this would be suitable for automated reporting or to add a screening step to divide cases into ones suitable for automated reporting, and those that are not, remains to be seen. The principles of using AI and automated bioinformatics pipelines and the techniques for validating these remain under development but are likely to play a pivotal role in the future of MRD testing.

### Challenges in the standardization of MRD

The wide variety of techniques available for measuring MRD makes it difficult to compare across different trials, unless the limit of detection and the reporting criteria for U-MRD is stated. **Table 8** outlines the MRD reporting level for the trials evaluated in this review, which have been standardized as per the ERIC (24) and iwCLL (17) criteria to U-MRD4, alongside some additional analyses to lower MRD levels. Even within the cutoff of U-MRD, the techniques available detect MRD to different sensitivities, and it is difficult to compare data with different techniques (summarized in **Tables 1**, **2**).

**Table 8:** MRD techniques and reporting of the trials.

Trial	MRD technique	MRD reporting
MURANO	PB both ASO-PCR and flow cytometry, BM flow cytometry	Neg when <U-MRD4
CLARITY	Flow cytometry	Neg when <U-MRD4
ELEVATE-TN	Flow cytometry	Neg when <U-MRD4
CLL14	ASO PCR	Neg when <U-MRD4
CAPTIVATE	Flow cytometry	Neg when <U-MRD4
GLOW	NGS (clonoSEQ), flow cytometry	Neg when <U-MRD4, additional analysis of MRD to 10^−5^
FLAIR	Flow cytometry	Neg when <U-MRD4
CLL13	Flow cytometry	Neg when <U-MRD4
GALACTIC	Flow cytometry	Neg when <U-MRD4

All flow cytometry techniques were multiparameter flow cytometry.

PB, peripheral blood; BM, bone marrow.

There is no recommendation to support the choice of one validated assay (flow cytometry and RQ-PCR) over the other. The choice of assay thus ultimately will depend on the reason for MRD monitoring and what test may be most accessible to the center.

The markers used in multicolor flow cytometry have also evolved over the years as more markers are identified allowing more accurate detection of residual disease (**Table 1**). As immunotherapy becomes an important factor in treatment regimens, there is concern that CD20 downregulation may make distinguishing CLL cells from normal B cells difficult. A study has shown a close correlation between MRD done by flow cytometry and RQ-PCR showing no effect of antibody treatment (9). However, further markers and combinations of markers have also been identified to account for this, e.g., CD81 in combination with CD19/CD22/CD5 (26). ERIC has been trying to standardize flow cytometry methods to assess MRD since 2007 to improve sensitivity and specificity but also to make results comparable between different laboratories. A core panel of six monoclonal antibodies (CD19, CD20, CD5, CD43, CD79b, CD81) developed by the ERIC group will be found on a high number of cases and is feasible in most laboratories (25) and helps standardize the process.

Emerging data on DNA-based monitoring, particularly the use of NGS (HTS), suggest that increasing the depth of MRD may be beneficial for trial and clinical outcomes (7, 35), although a consensus for validation of the technique is yet to be reached. There will also be a practical limitation of laboratory capacity (in terms of human, bench, financial, and computational resources) if MRD monitoring, particularly HTS, becomes adopted into routine practice. Computational tools could relieve some running and reporting burden for these assays but would require similar robust standardization and validation. A possible combined solution would be to screen samples with flow cytometry techniques and to elect samples undetectable on flow cytometry to more sensitive analysis, such as HTS.

### Clinical decisions with MRD results and patient’s experience

MRD already plays a very important prognostic marker in several hematological malignancies and is used to guide patient management. The use of MRD in CLL, however, is still largely confined to the trial settings presented here and is widely used as a marker for the efficacy of treatment while awaiting maturity of trial endpoints. In the context of chemoimmunotherapy where MRD was first used in this role, a longer progression-free survival was shown if U-MRD was achieved irrespective of clinical response (15, 76). A role for MRD in determining the duration of treatment, i.e., cessation of treatment with bone marrow U-MRD, was first demonstrated in the treatment with FCR (fludarabine, cyclophosphamide, rituximab). Patients who achieved BM U-MRD after 3 cycles of FCR and stopped had similar outcomes to those who completed all the planned cycles despite achieving U-MRD (77). A rise in MRD during post-treatment monitoring has also been shown to possibly anticipate clinical progression (78) and may allow earlier intervention.

BTK inhibitors are now frequently used as a single agent in the elderly or comorbid patients, or in the relapse setting, and are often given continuously until either progressive disease or unacceptable toxicity. The treatment goal is often disease control and not achieving U-MRD4 as long-term clinical responses are seen with continuous administration with increasing rates of CR over time in both frontline treatment and relapse/refractory disease treatment (79–81). Monitoring MRD levels may provide a means of potential safe treatment de-escalation or cessation but is potentially of limited value in single-agent BTKi regimens where U-MRD4 is expected to occur rarely. These regimens can still achieve good ORR and PFS rates even without obtaining a high U-MRD rate (59, 61). Conversely, MRD retains its potential utility when BTK inhibitors are administered in combination with another CLL-directed therapy with a high U-MRD4 rate, as part of a fixed-duration regimen or one with MRD-driven stopping rules. GLOW and CLL14 (fixed duration) are of particular relevance, reporting good outcomes in less fit cohorts (40, 52), in which a treatment-free period is likely to reduce the risk of potential toxicities associated with continuous treatment.

Patient counseling would be crucial if and when therapeutic decisions are made based on MRD results as negative results may give false reassurance while positive results may cause undue distress. Given that U-MRD can occur in the context of PR or CR/CRi, as demonstrated in CAPTIVATE (FD and MRD cohorts) (16, 41) and FLAIR (61), clarification of these terms and the relationship between them need to be addressed.

The increased use of novel agents in both first-line treatment and in relapsed/refractory disease means there is much less of a role for allogeneic stem cell transplant (SCT). However, this may still be an option albeit for a very small niche of patients, and MRD status in this context has been shown to be important in determining prognosis (82). As the use of CAR-T continues to increase, MRD may also provide a sensitive means of assessing disease status.

### MRD by disease compartment

As it is possible to have a discrepancy in some cases between the MRD result and disease status, it is important to consider the sites of disease that may not be represented by MRD assays done on PB or BM. The consensus recommendation for monitoring disease eradication in the trial setting at present is that both PB and BM should be assessed (16). However, there is a potential as assays continue to improve such as reducing the need for bone marrows, which involves an invasive and painful procedure. In the context of lymph node predominant disease or pure small lymphocytic lymphoma (SLL), where PB or BM MRD might not be representative of disease burden, MRD by techniques such as cell-free DNA may have a role in disease monitoring (70). The iwCLL response criteria are the same for either CLL or SLL treatment (17) and do not replace the clinical assessment of response. As such, MRD assessment is not a replacement for clinical assessment but another tool we can use in response assessment and prognostication.

### Role of traditional trial endpoints

In the clinical setting, more mature trial data are required to evaluate whether superior PFS translates into increased OS. In the context of multiple effective treatments now being available for a relatively elderly patient population, a difference in OS may be difficult to prove and may be confounded by comorbidities. PFS is a valuable outcome to report on the durability of treatment effect, but a more patient-orientated endpoint would be TTNT, although this will not necessarily correlate with OS. TTNT would evaluate the durability of fixed-duration treatment outcomes in terms of how long the patient remains treatment-free and report on the tolerability or duration of the effect of continuous (or MRD-driven intermittent) therapy. How MRD kinetics translate into traditional trial endpoints will also need to be considered as emerging evidence shows that these can vary according to disease characteristics, such as IGHV mutational status. The evolution and standardization of MRD monitoring in CLL has the potential to radically modify the management of patients, moving it closer to the management of other chronic hematological malignancies such as chronic myeloid leukemia, in which deep molecular response constitutes both an early therapeutic goal and a trigger for the initiation of subsequent lines of therapy.

### MRD use in fixed-duration and continuous therapy regimens

The trial regimens evaluated within this review have used fixed duration (8, 40, 41, 44, 52), continuous therapy (16, 38, 43, 57, 59), and MRD-based stopping rules (38, 43, 57) or randomizations (16, 45). It is unknown which of these approaches will be the most successful for balancing MRD effects that correlate with improved PFS or OS, against the side effects of treatment. Patient preference may well play into whether a fixed-duration or continuous treatment is chosen if outcomes are equivocal. Trial outcomes including PFS and MRD are not reported at consistent timepoints across trials, and the cohorts studied vary, making intertrial comparison challenging. However, it is worth noting that from the trials evaluated in the review, the PFS rates reported at ≥24 months for the BTKi- and/or venetoclax-containing arm are 81% to ≥95% (8, 16, 40, 41, 45, 52, 59), and the CIT arms are 38.8% to 49.5% (8, 40, 52, 59). When to measure MRD is also a point of debate for fixed-duration *versus* continuous therapy trial. If a fixed-duration treatment continues to the end of the regimen, unless there is clinical progression, there is an argument for measuring MRD after the end of treatment to predict the durability of response or inform ongoing or consolidation therapies if these becomes an option. Continuous treatment, especially regimens with stopping or pausing criteria, will require on-treatment MRD testing to inform treatment or monitoring decisions.

The key evaluation for MRD is whether U-MRD4 (or U-MRD5/6) leads to improved outcomes. The more mature data already suggest that this is the case, such as in MURANO where achievement of U-MRD4 correlated with superior OS and a lower MRD doubling time in the VR arm (46). In CLL14, patients with U-MRD4 at EOT had longer PFS compared with those with detectable MRD (40). Depth of MRD and the likely translation of this into the durability of response will be a key point to evaluate in the longer-term outcomes of fixed-duration *versus* continuous therapy, especially in a younger patient cohort in whom longevity is likely to be a priority.

### MRD in different genomic/biologic subgroups

Recent trial outcomes from GLOW, FLAIR, and CAPTIVATE FD highlight differential responses in cohorts with traditionally poor prognosis markers, uIGHV and TP53 disruptions. Again, the data are yet immature to determine if MRD differences by genetic subgroups will translate into PFS/OS/TTNT outcomes.

The IV arms of FLAIR and GLOW reported a superior rate of U-MRD4 for uIGHV patients in BM and PB, respectively. In FLAIR, 79.7% of uIGHV patients were MRD negative *versus* 56.4% with mIGHV at 2 years post-randomization. In GLOW at EOT+3, the uIGHV U-MRD4 rate was 59.7% *versus* 40.6% for mIGHV. GLOW also demonstrated that U-MRD4 was sustained in 80% of uIGHV patients and 76.9% of mIGHV patients (50). This shows that the current IGHV data obtained during the patient workup could be used to stratify those who will benefit the most if IV becomes used in clinical practice (4–6).

Data from the CAPTIVATE FD demonstrated, for TP53-disrupted patients, U-MRD4 81% in PB and 41% in BM after 12 months of IV. The differences in U-MRD4 between the TP53 wild-type and disrupted cohorts were not statistically significant (41). This result is expected, as BTKi and venetoclax therapy is already used preferentially in the context of TP53 disruption (4–6).

Subgroup analyses from larger trials may highlight which treatments have the most impact on certain groups but can lack the statistical power to be adopted into routine practice, which becomes ever more challenging as we subdivide already small cohorts further with emerging genomic findings.

## Concluding remarks

Evaluation of MRD has been transformational in the landscape of CLL clinical trial design and reporting. With an increasing number of MRD-directed endpoints, the next challenge is how MRD assessment will be integrated into routine clinical practice, which will also require evaluation from both patient benefit and health economic perspectives. Furthermore, different treatment types and combinations result in different outcomes concerning U-MRD, with respect to the number of patients achieving this status, the depth of MRD obtained, and the biological subgroups undergoing treatment. Importantly, the relevant threshold for MRD remains a matter of debate, and it remains to be demonstrated that deeper MRD necessarily predicts a better clinical outcome in all clinical situations. At present, MRD has been linked to disease prognosis and used to tailor treatment duration, but it remains to be demonstrated whether MRD-driven therapeutic decisions will always be clinically beneficial or sufficient to challenge the traditional CLL treatment paradigms.

## Author contributions

FA and GH wrote the paper. M-CN, PP, and MT reviewed and edited the manuscript. All authors read and approved the manuscript.

## References

[1] Fischer K Bahlo J Fink AM Goede V Herling CD Cramer P. Long-term remissions after fcr chemoimmunotherapy in previously untreated patients with cll: Updated results of the Cll8 trial. Blood (2016) 127(2):208–15. doi: 10.1182/blood-2015-06-651125 26486789

[2] Eichhorst B Fink AM Bahlo J Busch R Kovacs G Maurer C. First-line chemoimmunotherapy with bendamustine and rituximab versus fludarabine, cyclophosphamide, and rituximab in patients with advanced chronic lymphocytic leukaemia (Cll10): An international, open-label, randomised, phase 3, non-inferiority trial. Lancet Oncol (2016) 17(7):928–42. doi: 10.1016/S1470-2045(16)30051-1 27216274

[3] Goede V Fischer K Busch R Engelke A Eichhorst B Wendtner CM. Obinutuzumab plus chlorambucil in patients with cll and coexisting conditions. N Engl J Med (2014) 370(12):1101–10. doi: 10.1056/NEJMoa1313984 24401022

[4] Walewska R Parry-Jones N Eyre TA Follows G Martinez-Calle N McCarthy H. Guideline for the treatment of chronic lymphocytic leukaemia. Br J Haematol (2022) 197(5):544–57. doi: 10.1111/bjh.18075 35313007

[5] Eichhorst B Robak T Montserrat E Ghia P Niemann CU Kater AP. Chronic lymphocytic leukaemia: Esmo clinical practice guidelines for diagnosis, treatment and follow-up. Ann Oncol (2021) 32(1):23–33. doi: 10.1016/j.annonc.2020.09.019 33091559

[6] Wierda W BJ Abramson J Awan F Bilgrami S Bociek G. Nccn guidelines version 1.2023 chronic lymphocytic Leukemia/Small lymphocytic lymphoma (2022). Available at: https://www.nccn.org/professionals/physician_gls/pdf/cll.pdf.

[7] Letestu R Dahmani A Boubaya M Baseggio L Campos L Chatelain B. Prognostic value of high-sensitivity measurable residual disease assessment after front-line chemoimmunotherapy in chronic lymphocytic leukemia. Leukemia (2021) 35(6):1597–609. doi: 10.1038/s41375-020-01009-z 32934355

[8] Kater AP Seymour JF Hillmen P Eichhorst B Langerak AW Owen C. Fixed duration of venetoclax-rituximab in Relapsed/Refractory chronic lymphocytic leukemia eradicates minimal residual disease and prolongs survival: Post-treatment follow-up of the murano phase iii study. J Clin Oncol (2019) 37(4):269–77. doi: 10.1200/JCO.18.01580 30523712

[9] Böttcher S Stilgenbauer S Busch R Bruggemann M Raff T Pott C. Standardized mrd flow and aso igh rq-pcr for mrd quantification in cll patients after rituximab-containing immunochemotherapy: A comparative analysis. Leukemia (2009) 23(11):2007–17. doi: 10.1038/leu.2009.140 19641522

[10] Kovacs G Robrecht S Fink AM Bahlo J Cramer P von Tresckow J. Minimal residual disease assessment improves prediction of outcome in patients with chronic lymphocytic leukemia (Cll) who achieve partial response: Comprehensive analysis of two phase iii studies of the German cll study group. J Clin Oncol (2016) 34(31):3758–65. doi: 10.1200/JCO.2016.67.1305 27573660

[11] Dimier N Delmar P Ward C Morariu-Zamfir R Fingerle-Rowson G Bahlo J. A model for predicting effect of treatment on progression-free survival using mrd as a surrogate end point in cll. Blood (2018) 131(9):955–62. doi: 10.1182/blood-2017-06-792333 29255066

[12] Thompson PA Tam CS O'Brien SM Wierda WG Stingo F Plunkett W. Fludarabine, cyclophosphamide, and rituximab treatment achieves long-term disease-free survival in ighv-mutated chronic lymphocytic leukemia. Blood (2016) 127(3):303–9. doi: 10.1182/blood-2015-09-667675 PMC4760129 26492934

[13] Thompson PA Srivastava J Peterson C Strati P Jorgensen JL Hether T. Minimal residual disease undetectable by next-generation sequencing predicts improved outcome in cll after chemoimmunotherapy. Blood (2019) 134(22):1951–9. doi: 10.1182/blood.2019001077 PMC6887113 31537528

[14] Kwok M Rawstron AC Varghese A Evans PA O'Connor SJ Doughty C. Minimal residual disease is an independent predictor for 10-year survival in cll. Blood (2016) 128(24):2770–3. doi: 10.1182/blood-2016-05-714162 27697770

[15] Böttcher S Ritgen M Fischer K Stilgenbauer S Busch RM Fingerle-Rowson G. Minimal residual disease quantification is an independent predictor of progression-free and overall survival in chronic lymphocytic leukemia: A multivariate analysis from the randomized gcllsg Cll8 trial. J Clin Oncol (2012) 30(9):980–8. doi: 10.1200/JCO.2011.36.9348 22331940

[16] Wierda WG Allan JN Siddiqi T Kipps TJ Opat S Tedeschi A. Ibrutinib plus venetoclax for first-line treatment of chronic lymphocytic leukemia: Primary analysis results from the minimal residual disease cohort of the randomized phase ii captivate study. J Clin Oncol (2021) 39(34):3853–65. doi: 10.1200/JCO.21.00807 PMC8713593 34618601

[17] Hallek M Cheson BD Catovsky D Caligaris-Cappio F Dighiero G Dohner H. Iwcll guidelines for diagnosis, indications for treatment, response assessment, and supportive management of cll. Blood (2018) 131(25):2745–60. doi: 10.1182/blood-2017-09-806398 29540348

[18] (CHMP). Summary of opinion (Post authorisation) - brukinsa (2022). Available at: https://www.ema.europa.eu/en/documents/smop/chmp-post-authorisation-summary-positive-opinion-brukinsa-ii-03_en.pdf.

[19] Jones JA Byrd JC. How will b-Cell-Receptor-Targeted therapies change future cll therapy? Blood (2014) 123(10):1455–60. doi: 10.1182/blood-2013-09-453092 PMC3945859 24394667

[20] Tobinai K Klein C Oya N Fingerle-Rowson G. A review of obinutuzumab (Ga101), a novel type ii anti-Cd20 monoclonal antibody, for the treatment of patients with b-cell malignancies. Adv Ther (2017) 34(2):324–56. doi: 10.1007/s12325-016-0451-1 PMC5331088 28004361

[21] Oscier D Dearden C Eren E Fegan C Follows G Hillmen P. Guidelines on the diagnosis, investigation and management of chronic lymphocytic leukaemia. Br J Haematol (2012) 159(5):541–64. doi: 10.1111/bjh.12067 23057493

[22] Wierda WG Rawstron A Cymbalista F Badoux X Rossi D Brown JR. Measurable residual disease in chronic lymphocytic leukemia: Expert review and consensus recommendations. Leukemia (2021) 35(11):3059–72. doi: 10.1038/s41375-021-01241-1 PMC8550962 34168283

[23] Letestu R CG Cartron G Lepretre S Le Garff-Tavernier M Solly F Campos L. Minimal residual disease (Mrd) by 8-color flow cytometry (Flow-mrd) and igh clonospecific quantitative pcr (Aso rqpcr) reached similar performances for evaluation of cll treatment in a phase ii clinical trial: Cross validation of the methods. Blood (2014) 124:3307. doi: 10.1182/blood.V124.21.3307.3307

[24] Rawstron AC Bottcher S Letestu R Villamor N Fazi C Kartsios H. Improving efficiency and sensitivity: European research initiative in cll (Eric) update on the international harmonised approach for flow cytometric residual disease monitoring in cll. Leukemia (2013) 27(1):142–9. doi: 10.1038/leu.2012.216 23041722

[25] Rawstron AC Fazi C Agathangelidis A Villamor N Letestu R Nomdedeu J. A complementary role of multiparameter flow cytometry and high-throughput sequencing for minimal residual disease detection in chronic lymphocytic leukemia: An European research initiative on cll study. Leukemia (2016) 30(4):929–36. doi: 10.1038/leu.2015.313 PMC4832072 26639181

[26] Rawstron AC Villamor N Ritgen M Bottcher S Ghia P Zehnder JL. International standardized approach for flow cytometric residual disease monitoring in chronic lymphocytic leukaemia. Leukemia (2007) 21(5):956–64. doi: 10.1038/sj.leu.2404584 17361231

[27] Dowling AK Liptrot SD O'Brien D Vandenberghe E. Optimization and validation of an 8-color single-tube assay for the sensitive detection of minimal residual disease in b-cell chronic lymphocytic leukemia detected *Via* flow cytometry. Lab Med (2016) 47(2):103–11. doi: 10.1093/labmed/lmw006 27069028

[28] Sartor MM Gottlieb DJ. A single tube 10-color flow cytometry assay optimizes detection of minimal residual disease in chronic lymphocytic leukemia. Cytomet B Clin Cytom (2013) 84(2):96–103. doi: 10.1002/cyto.b.21067 23283845

[29] Goshaw JM Gao Q Wardrope J Dogan A Roshal M. 14-color single tube for flow cytometric characterization of Cd5+ b-lpds and high sensitivity automated minimal residual disease quantitation of Cll/Sll. Cytomet B Clin Cytom (2021) 100(4):509–18. doi: 10.1002/cyto.b.21953 PMC9448444 32896973

[30] Bazinet A Rys RN Barry A Greenwood CMT Young YK Mendoza A. A 10-color flow cytometry panel for diagnosis and minimal residual disease in chronic lymphocytic leukemia. Leuk Lymphoma (2021) 62(10):2352–9. doi: 10.1080/10428194.2021.1919658 34020575

[31] van Dongen JJ Lhermitte L Bottcher S Almeida J van der Velden VH Flores-Montero J. Euroflow antibody panels for standardized n-dimensional flow cytometric immunophenotyping of normal, reactive and malignant leukocytes. Leukemia (2012) 26(9):1908–75. doi: 10.1038/leu.2012.120 PMC3437410 22552007

[32] Sanchez R Ayala R Martinez-Lopez J. Minimal residual disease monitoring with next-generation sequencing methodologies in hematological malignancies. Int J Mol Sci (2019) 20(11):2832. doi: 10.3390/ijms20112832 31185671 PMC6600313

[33] Logan AC Gao H Wang C Sahaf B Jones CD Marshall EL. High-throughput vdj sequencing for quantification of minimal residual disease in chronic lymphocytic leukemia and immune reconstitution assessment. Proc Natl Acad Sci U.S.A. (2011) 108(52):21194–9. doi: 10.1073/pnas.1118357109 PMC3248502 22160699

[34] Raponi S Della Starza I De Propris MS Del Giudice I Mauro FR Marinelli M. Minimal residual disease monitoring in chronic lymphocytic leukaemia patients. a comparative analysis of flow cytometry and aso igh rq-pcr. Br J Haematol (2014) 166(3):360–8. doi: 10.1111/bjh.12887 24735016

[35] Hengeveld PJ van der Klift MY Kolijn PM Davi F Kavelaars FG de Jonge E. Detecting measurable residual disease beyond 10-4 through an ighv leader-based ngs approach improves prognostic stratification in cll. Blood (2022) 141 (5) 519–28. doi: 10.1182/blood.2022017411 36084320

[36] van der Velden VH Hochhaus A Cazzaniga G Szczepanski T Gabert J van Dongen JJ. Detection of minimal residual disease in hematologic malignancies by real-time quantitative pcr: Principles, approaches, and laboratory aspects. Leukemia (2003) 17(6):1013–34. doi: 10.1038/sj.leu.2402922 12764363

[37] Seymour JF Kipps TJ Eichhorst B Hillmen P D'Rozario J Assouline S. Venetoclax-rituximab in relapsed or refractory chronic lymphocytic leukemia. N Engl J Med (2018) 378(12):1107–20. doi: 10.1056/NEJMoa1713976 29562156

[38] Hillmen P Rawstron AC Brock K Munoz-Vicente S Yates FJ Bishop R. Ibrutinib plus venetoclax in Relapsed/Refractory chronic lymphocytic leukemia: The clarity study. J Clin Oncol (2019) 37(30):2722–9. doi: 10.1200/JCO.19.00894 PMC6879312 31295041

[39] Sharman JP Egyed M Jurczak W Skarbnik A Pagel JM Flinn IW. Acalabrutinib with or without obinutuzumab versus chlorambucil and obinutuzmab for treatment-naive chronic lymphocytic leukaemia (Elevate tn): A randomised, controlled, phase 3 trial. Lancet (2020) 395(10232):1278–91. doi: 10.1016/S0140-6736(20)30262-2 PMC8151619 32305093

[40] Al-Sawaf O Zhang C Tandon M Sinha A Fink AM Robrecht S. Venetoclax plus obinutuzumab versus chlorambucil plus obinutuzumab for previously untreated chronic lymphocytic leukaemia (Cll14): Follow-up results from a multicentre, open-label, randomised, phase 3 trial. Lancet Oncol (2020) 21(9):1188–200. doi: 10.1016/S1470-2045(20)30443-5 32888452

[41] TamCSAllanJNSiddiqiTKippsTJJacobsROpatS. Fixed-duration ibrutinib plus venetoclax for first-line treatment of cll: Primary analysis of the captivate fd cohort. Blood (2022) 139(22):3278–89. doi: 10.1182/blood.2021014488 35196370

[42] Kater Arnon P Owen C Moreno C Follows G Munir T Levin M-D. Fixed-duration ibrutinib-venetoclax in patients with chronic lymphocytic leukemia and comorbidities. NEJM Evidence (2022) 1(7):EVIDoa2200006. doi: 10.1056/EVIDoa2200006 38319255

[43] Howard DR Hockaday A Brown JM Gregory WM Todd S Munir T. A platform trial in practice: Adding a new experimental research arm to the ongoing confirmatory flair trial in chronic lymphocytic leukaemia. Trials (2021) 22(1):38. doi: 10.1186/s13063-020-04971-2 33419469 PMC7792072

[44] Eichhorst B NC Niemann C Kater AP Furstenau M Von Tresckow J Zhang C. A randomised phase iii study of venetoclax-based time-limited combination treatments (Rve, gve, give) vs standard chemoimmunotherpay (Cit: Fcr/Br) in frontline chronic lymphocytic leukaemia (Cll) of fit patients: First Co-primary endpoint analysis of the international intergroup gaia (Cll13) trial. Blood (2021) 138:1. doi: 10.1182/blood-2021-146161 34236425

[45] Munir T Emmerson J Hockaday A Oughton JB Howard D Phillips D. Obinutuzumab as consolidation after chemo-immunotherapy: Results of the uk national cancer research institute phase Ii/Iii galactic trial. Br J Haematol (2022) 199(5):707–19. doi: 10.1111/bjh.18427 PMC9805170 36017875

[46] Seymour JF Kipps TJ Eichhorst BF D'Rozario J Owen CJ Assouline S. Enduring undetectable mrd and updated outcomes in Relapsed/Refractory cll after fixed-duration venetoclax-rituximab. Blood (2022) 140(8):839–50. doi: 10.1182/blood.2021015014 PMC9412011 35605176

[47] Munir T Cherrill L-R Webster N Dalal S Boucher RH Sankhalpara C. (2022). Mrd4 eradication at 6 months and early clearance of mrd with combination of ibrutinib plus venetoclax results in sustained clinical and mrd responses: Exploratory analysis of the blood cancer uk tap clarity study. Blood 140 :222–3.

[48] Chanan-Khan A Cramer P Demirkan F Fraser G Silva RS Grosicki S. Ibrutinib combined with bendamustine and rituximab compared with placebo, bendamustine, and rituximab for previously treated chronic lymphocytic leukaemia or small lymphocytic lymphoma (Helios): A randomised, double-blind, phase 3 study. Lancet Oncol (2016) 17(2):200–11. doi: 10.1016/S1470-2045(15)00465-9 26655421

[49] Robak T Dmoszynska A Solal-Celigny P Warzocha K Loscertales J Catalano J. Rituximab plus fludarabine and cyclophosphamide prolongs progression-free survival compared with fludarabine and cyclophosphamide alone in previously treated chronic lymphocytic leukemia. J Clin Oncol (2010) 28(10):1756–65. doi: 10.1200/JCO.2009.26.4556 20194844

[50] Niemann CU Munir T Moreno C Owen C Follows G Benjamini O. (2022). Residual disease kinetics among patients with high-risk factors treated with first-line fixed-duration ibrutinib plus venetoclax (Ibr+Ven) versus chlorambucil plus obinutuzumab (Clb+O): The glow study. Blood 140:228–30.

[51] Munir T Pitchford A Bloor A Pettitt A Patten PEM Forconi F. (2022). Combination of ibrutinib plus venetoclax with mrd-driven duration of treatment results in a higher rate of mrd negativity in ighv unmutated than mutated cll: Updated interim analysis of flair study. Blood 140 :231–3.

[52] Munir T Moreno C Owen C Follows GA Benjamini O Janssens A. (2021). First prospective data on minimal residual disease (Mrd) outcomes after fixed-duration ibrutinib plus venetoclax (Ibr+Ven) versus chlorambucil plus obinutuzumab (Clb+O) for first-line treatment of cll in elderly or unfit patients: The glow study. Blood 138:70

[53] Mayer RL Schwarzmeier JD Gerner MC Bileck A Mader JC Meier-Menches SM. Proteomics and metabolomics identify molecular mechanisms of aging potentially predisposing for chronic lymphocytic leukemia. Mol Cell Proteomics (2018) 17(2):290–303. doi: 10.1074/mcp.RA117.000425 29196338 PMC5795392

[54] Byrd JC Furman RR Coutre SE Flinn IW Burger JA Blum KA. Targeting btk with ibrutinib in relapsed chronic lymphocytic leukemia. N Engl J Med (2013) 369(1):32–42. doi: 10.1056/NEJMoa1215637 23782158 PMC3772525

[55] O'Brien S Furman RR Coutre SE Sharman JP Burger JA Blum KA. Ibrutinib as initial therapy for elderly patients with chronic lymphocytic leukaemia or small lymphocytic lymphoma: An open-label, multicentre, phase 1b/2 trial. Lancet Oncol (2014) 15(1):48–58. doi: 10.1016/S1470-2045(13)70513-8 24332241 PMC4134524

[56] Hallek M Cheson BD Catovsky D Caligaris-Cappio F Dighiero G Dohner H. Guidelines for the diagnosis and treatment of chronic lymphocytic leukemia: A report from the international workshop on chronic lymphocytic leukemia updating the national cancer institute-working group 1996 guidelines. Blood (2008) 111(12):5446–56. doi: 10.1182/blood-2007-06-093906 PMC2972576 18216293

[57] Collett L Howard DR Munir T McParland L Oughton JB Rawstron AC. Assessment of ibrutinib plus rituximab in front-line cll (Flair trial): Study protocol for a phase iii randomised controlled trial. Trials (2017) 18(1):387. doi: 10.1186/s13063-017-2138-6 28830517 PMC5568356

[58] Burger JA Sivina M Jain N Kim E Kadia T Estrov Z. Randomized trial of ibrutinib vs ibrutinib plus rituximab in patients with chronic lymphocytic leukemia. Blood (2019) 133(10):1011–9. doi: 10.1182/blood-2018-10-879429 PMC6405333 30530801

[59] Sharman JP Egyed M Jurczak W Skarbnik A Pagel JM Flinn IW. Efficacy and safety in a 4-year follow-up of the elevate-tn study comparing acalabrutinib with or without obinutuzumab versus obinutuzumab plus chlorambucil in treatment-naive chronic lymphocytic leukemia. Leukemia (2022) 36(4):1171–5. doi: 10.1038/s41375-021-01485-x PMC8979808 34974526

[60] Hillmen P Pitchford A Bloor A Broom A Young M Kennedy B. Ibrutinib plus rituximab is superior to fcr in previously untreated cll: Results of the phase iii ncri flair trial. Blood (2021) 138:642. doi: 10.1182/blood-2021-152319

[61] Hillmen P Pitchford A Bloor A Pettitt A Patten P Forconi F. (2022). The combination of ibrutinib plus venetoclax results in a high rate of mrd negativity in previously untreated cll: The results of the planned interim analysis of the phase 3 ncri flair trial. HemaSphere 6 :46–7.

[62] Al-Sawaf O Fischer K Herling CD Ritgen M Bottcher S Bahlo J. Alemtuzumab consolidation in chronic lymphocytic leukaemia: A phase I/Ii multicentre trial. Eur J Haematol (2017) 98(3):254–62. doi: 10.1111/ejh.12825 27862308

[63] Lin TS Donohue KA Byrd JC Lucas MS Hoke EE Bengtson EM. Consolidation therapy with subcutaneous alemtuzumab after fludarabine and rituximab induction therapy for previously untreated chronic lymphocytic leukemia: Final analysis of calgb 10101. J Clin Oncol (2010) 28(29):4500–6. doi: 10.1200/JCO.2010.29.7978 PMC2988639 20697069

[64] Varghese AM Howard DR Pocock C Rawstron AC Follows G McCarthy H. Eradication of minimal residual disease improves overall and progression-free survival in patients with chronic lymphocytic leukaemia, evidence from ncrn Cll207: A phase ii trial assessing alemtuzumab consolidation. Br J Haematol (2017) 176(4):573–82. doi: 10.1111/bjh.14342 28032335

[65] Wendtner CM Ritgen M Schweighofer CD Fingerle-Rowson G Campe H Jager G. Consolidation with alemtuzumab in patients with chronic lymphocytic leukemia (Cll) in first remission–experience on safety and efficacy within a randomized multicenter phase iii trial of the German cll study group (Gcllsg). Leukemia (2004) 18(6):1093–101. doi: 10.1038/sj.leu.2403354 15071604

[66] Galimberti S Balducci S Guerrini F Del Re M Cacciola R. Digital droplet pcr in hematologic malignancies: A new useful molecular tool. Diagnostics (2022) 12(6):1305. doi: 10.3390/diagnostics12061305 35741115 PMC9221914

[67] Frazzi R Bizzarri V Albertazzi L Cusenza VY Coppolecchia L Luminari S. Droplet digital pcr is a sensitive tool for the detection of Tp53 deletions and point mutations in chronic lymphocytic leukaemia. Br J Haematol (2020) 189(2):e49–52. doi: 10.1111/bjh.16442 31943144

[68] Minervini A Francesco Minervini C Anelli L Zagaria A Casieri P Coccaro N. Droplet digital pcr analysis of Notch1 gene mutations in chronic lymphocytic leukemia. Oncotarget (2016) 7(52):86469–79. doi: 10.18632/oncotarget.13246 PMC5349927 27835908

[69] Appay R Fina F Barets D Gallardo C Nanni-Metellus I Scavarda D. Multiplexed droplet digital pcr assays for the simultaneous screening of major genetic alterations in tumors of the central nervous system. Front Oncol (2020) 10:579762. doi: 10.3389/fonc.2020.579762 33282733 PMC7689380

[70] Yeh P Hunter T Sinha D Ftouni S Wallach E Jiang D. Circulating tumour DNA reflects treatment response and clonal evolution in chronic lymphocytic leukaemia. Nat Commun (2017) 8:14756. doi: 10.1038/ncomms14756 28303898 PMC5357854

[71] Shanmuganathan N Branford S. Multiplex technologies for the assessment of minimal residual disease and low-level mutation detection in leukaemia: Mass spectrometry versus next-generation sequencing. Br J Haematol (2022) 196(1):19–30. doi: 10.1111/bjh.17623 34124782

[72] Gadalla R Noamani B MacLeod BL Dickson RJ Guo M Xu W. Validation of cytof against flow cytometry for immunological studies and monitoring of human cancer clinical trials. Front Oncol (2019) 9:415. doi: 10.3389/fonc.2019.00415 31165047 PMC6534060

[73] Nowicka M Krieg C Crowell HL Weber LM Hartmann FJ Guglietta S. Cytof workflow: Differential discovery in high-throughput high-dimensional cytometry datasets. F1000Res (2017) 6:748. doi: 10.12688/f1000research.11622.3 28663787 PMC5473464

[74] Salama ME Otteson GE Camp JJ Seheult JN Jevremovic D Holmes DR. Artificial intelligence enhances diagnostic flow cytometry workflow in the detection of minimal residual disease of chronic lymphocytic leukemia. Cancers (2022) 14(10):2537. doi: 10.3390/cancers14102537 35626140 PMC9139233

[75] Nguyen P NV Came N Westerman D. Unsupervised computational detection of measurable residual disease in chronic lymphocytic leukaemia. Pathology (2022) 54:S30–S1. doi: 10.1016/j.pathol.2021.12.101

[76] Fischer K Cramer P Busch R Bottcher S Bahlo J Schubert J. Bendamustine in combination with rituximab for previously untreated patients with chronic lymphocytic leukemia: A multicenter phase ii trial of the German chronic lymphocytic leukemia study group. J Clin Oncol (2012) 30(26):3209–16. doi: 10.1200/JCO.2011.39.2688 22869884

[77] Strati P Keating MJ O'Brien SM Burger J Ferrajoli A Jain N. Eradication of bone marrow minimal residual disease may prompt early treatment discontinuation in cll. Blood (2014) 123(24):3727–32. doi: 10.1182/blood-2013-11-538116 PMC4067501 24705492

[78] Thompson PA Peterson CB Strati P Jorgensen J Keating MJ O'Brien SM. Serial minimal residual disease (Mrd) monitoring during first-line fcr treatment for cll may direct individualized therapeutic strategies. Leukemia (2018) 32(11):2388–98. doi: 10.1038/s41375-018-0132-y PMC6192870 29769624

[79] Barr PM Robak T Owen C Tedeschi A Bairey O Bartlett NL. Sustained efficacy and detailed clinical follow-up of first-line ibrutinib treatment in older patients with chronic lymphocytic leukemia: Extended phase 3 results from resonate-2. Haematologica (2018) 103(9):1502–10. doi: 10.3324/haematol.2018.192328 PMC6119145 29880603

[80] Brown JR Hillmen P O'Brien S Barrientos JC Reddy NM Coutre SE. Extended follow-up and impact of high-risk prognostic factors from the phase 3 resonate study in patients with previously treated Cll/Sll. Leukemia (2018) 32(1):83–91. doi: 10.1038/leu.2017.175 28592889 PMC5770586

[81] O'Brien S Jones JA Coutre SE Mato AR Hillmen P Tam C. Ibrutinib for patients with relapsed or refractory chronic lymphocytic leukaemia with 17p deletion (Resonate-17): A phase 2, open-label, multicentre study. Lancet Oncol (2016) 17(10):1409–18. doi: 10.1016/S1470-2045(16)30212-1 27637985

[82] Dreger P Schnaiter A Zenz T Bottcher S Rossi M Paschka P. Tp53, Sf3b1, and Notch1 mutations and outcome of allotransplantation for chronic lymphocytic leukemia: Six-year follow-up of the gcllsg Cll3x trial. Blood (2013) 121(16):3284–8. doi: 10.1182/blood-2012-11-469627 23435461

